# Monthly Record of Deaths in the City of Buffalo

**Published:** 1857-07

**Authors:** C. L. Dayton

**Affiliations:** Health Physician


ART. V.—*Report of Mortality in Buffalo for the month of May*, 1857. By C. L. DAYTON, M. D., Health Physician.

| DISEASES. | No. | Males. | Females. |
|---|---|---|---|
| Asthma, | 1 | | 1 |
| Aneurism, | 1 | 1 | |
| Accident, | 1 | 1 | |
| Apoplexia, | 2 | 2 | |
| Asphyxia Immersorum, | 1 | 1 | |
| Cephalitis, | 3 | 1 | 2 |
| Croup, | 4 | 4 | |
| Dentitio, | 2 | 1 | 1 |
| Diarrhœa Chronica, | 3 | | 3 |
| Dysenteria, | 1 | | 1 |
| Debilitas Senilis, | 6 | 3 | 3 |
| Epilepsy, | 1 | 1 | |
| Erysipelas, | 1 | 1 | |
| Eclampsia, | 19 | 11 | 8 |
| " Puerperarum, | 1 | | 1 |
| Enteritis et Perforatio Intestinalis, | 1 | 1 | |
| Febris Typhoid, | 6 | 3 | 3 |
| " Scarlatina et sequelæ, | 18 | 11 | 7 |
| " Nervosa, | 2 | 1 | 1 |
| " Puerperarum, | 1 | | 1 |
| " Intermittent, | 1 | 1 | |
| Hydrocephalus, | 6 | 4 | 2 |
| Hepatitis, | 1 | | 1 |
| Hyperæmia Pulmonalis, | 3 | 2 | 1 |
| " Cerebralis, | 2 | 2 | |
| Ignotus, | 28 | 18 | 10 |
| Intemperance, | 2 | 1 | 1 |
| Morbus Cordis, | 5 | 2 | 3 |
| Mania Puerperarum, | 1 | | 1 |
| Marasmus, | 5 | 4 | 1 |
| Paralysis, | 2 | 1 | 1 |
| Pertussis, | 1 | 1 | |
| Phlebitis, | 1 | | 1 |
| Pyæmia, | 2 | | 2 |
| Pneumonitis, | 15 | 11 | 4 |
| Peritonitis, | 2 | 1 | 1 |
| Scrophula, | 2 | 1 | 1 |
| Still-born, | 2 | 2 | |
| Suicide, | 2 | 1 | 1 |
| Syphilis Congenital, | 2 | 1 | 1 |
| Tonsilitis Chronica, | 1 | | 1 |
| Tuberculosis Pulmonalis, | 20 | 11 | 9 |
| Variola, | 1 | | 1 |
| Total, | 182 | | |

### SEXES.

| | |
|---|---|
| Males, | 107 |
| Females, | 75 |
| Sex not given, | 0 |
| Total, | 183 |

### AGES.

| | | | |
|---|---|---|---|
| Still-born, | 2 | Between 20 years and 30 years, | 16 |
| 1 day, | 3 | " 30 " " 40 " | 13 |
| 1 day and 30 days, | 12 | " 40 " " 50 " | 5 |
| Between 1 month and 6 months, | 23 | " 50 " " 60 " | 11 |
| " 6 months and 12 " | 25 | " 60 " " 70 " | 5 |
| " 1 year " 3 years, | 28 | " 70 " " 80 " | 4 |
| " 3 " " 5 " | 15 | " 80 " " 90 " | 1 |
| " 5 " " 10 " | 6 | " 90 " " 100 " | 0 |
| " 10 " " 20 " | 13 | " 100 " | 0 |
| Total number under 10 years, | 114 | Total number over 10 years, | 68 |
| Ages not given, | 0 | Total, | 182 |

### NATIVITIES.

| | | | |
|---|---|---|---|
| American, (white,) | 95 | Welsh, | 1 |
| " (colored,) | 3 | French, | 4 |
| German, | 35 | Scotch, | 2 |
| Irish, | 18 | Holland, | 1 |
| English, | 4 | Country not given, | 18 |
| Canadian, | 1 | | |
| Total, | | | 182 |

